# Distinct topographic-anatomical patterns in primary and secondary brain tumors and their therapeutic potential

**DOI:** 10.1007/s11060-020-03574-w

**Published:** 2020-07-08

**Authors:** Kevin Akeret, Victor E. Staartjes, Flavio Vasella, Carlo Serra, Jorn Fierstra, Marian Christoph Neidert, Luca Regli, Niklaus Krayenbühl

**Affiliations:** 1 Department of Neurosurgery, Clinical Neuroscience Center, University Hospital Zurich, University of Zurich, Zurich, Switzerland; 2 Division of Pediatric Neurosurgery, University Children’s Hospital, Zurich, Switzerland

**Keywords:** Anatomy, Anatomically tailored supratotal resection, Pathoclisis, Topography, Ventriculo-cortical radial unit

## Abstract

**Purpose:**

Understanding the topographic-anatomical patterns of brain tumors has the potential to improve our pathophysiological understanding and may allow for anatomical tailoring of surgery and radiotherapy. This study analyzed topographic-anatomical patterns underlying neuroepithelial tumors, primary CNS lymphoma and metastases.

**Methods:**

Any histologically confirmed supra- or infratentorial parenchymal neoplasia of one institution over a 4-year period was included. Using high-resolution magnetic resonance imaging data, a detailed analysis of the topographic-anatomical tumor features was performed. Differences between neuroepithelial tumors, primary central nervous system lymphoma (PCNSL) and metastases were assessed using pairwise comparisons adjusted for multiple testing, upon significance of the omnibus test.

**Results:**

Based on image analysis of 648 patients—419 (65%) neuroepithelial tumors, 28 (5%) PCNSL and 201 (31%) metastases—entity-specific topographic-anatomical patterns were identified. Neuroepithelial tumors showed a radial ventriculo-cortical orientation, inconsistent with the current belief of a growth along white matter tracts, whereas the pattern in PCNSL corresponded to a growth along such. Metastases preferentially affected the cortex and subcortical white matter of large arteries’ terminal supply areas. This study provides a comprehensive anatomical description of the topography of NT, PCNSL and metastases intended to serve as a topographic reference for clinicians and neuroscientists.

**Conclusions:**

The identified distinct anatomical patterns provide evidence for a specific interaction between tumor and anatomical structures, following a pathoclitic concept. Understanding differences in their anatomical behavior has the potential to improve our pathophysiological understanding and to tailor therapy of brain tumors.

**Electronic supplementary material:**

The online version of this article (10.1007/s11060-020-03574-w) contains supplementary material, which is available to authorized users.

## Introduction

Classification and treatment of neuroepithelial tumors (NT), primary central nervous system lymphoma (PCNSL) and brain metastases depend largely on microscopic and molecular characteristics [1]. Macroscopic properties of brain tumors, such as topographic-anatomical patterns, are not taken into account and, surprisingly, have never been described in detail. This is despite the fact that the brain with its complex micro- and macroarchitecture, determined by the underlying phylo- and ontogenesis, is known to be not uniformly affected by disease processes. Pathoclisis [2], i.e. anatomically selective brain vulnerability, has been described for genetic [3], inflammatory [4], infectious [5], degenerative [6], metabolic [7], toxic [8] and vascular [9] brain pathologies as well as epilepsy [10]. Similarly, neoplasms may originate from distinct brain regions and grow in defined spatial patterns, representing a specific interaction between the pathology and anatomical structures. As the identification of differences in the anatomical behavior of brain tumors has the potential to enhance our pathophysiological understanding and to improve their therapy, it was the aim of this study to provide a detailed description of the topographic-anatomical patterns of NT, PCNSL and metastases.

## Methods

### Study population

Prospectively and consecutively collected epidemiologic, imaging and histopathological data of every patient having undergone brain tumor surgery at our tertiary care hospital over a 4-year period (April 2009 to March 2013) was reviewed. The following were criteria for the inclusion of patients in the study: (i) a histopathologically confirmed NT, PCNSL or metastases after biopsy or resection, (ii) an intraparenchymal encephalic tumor location (supra-, infratentorial or both), (iii) first diagnosis without any pretreatment, and (iv) availability of complete standardized preoperative high resolution magnetic resonance imaging (MRI) data (technical details in Supplementary methods). Patients with (i) recurrent or pretreated brain tumors, (ii) extraaxial or spinal tumor location, (iii) inconclusive histopathological results, (iv) MRI data not fulfilling the mentioned requirements, or (v) any history of previous cranial surgery were excluded.

### Data collection

**Fig. 1:**
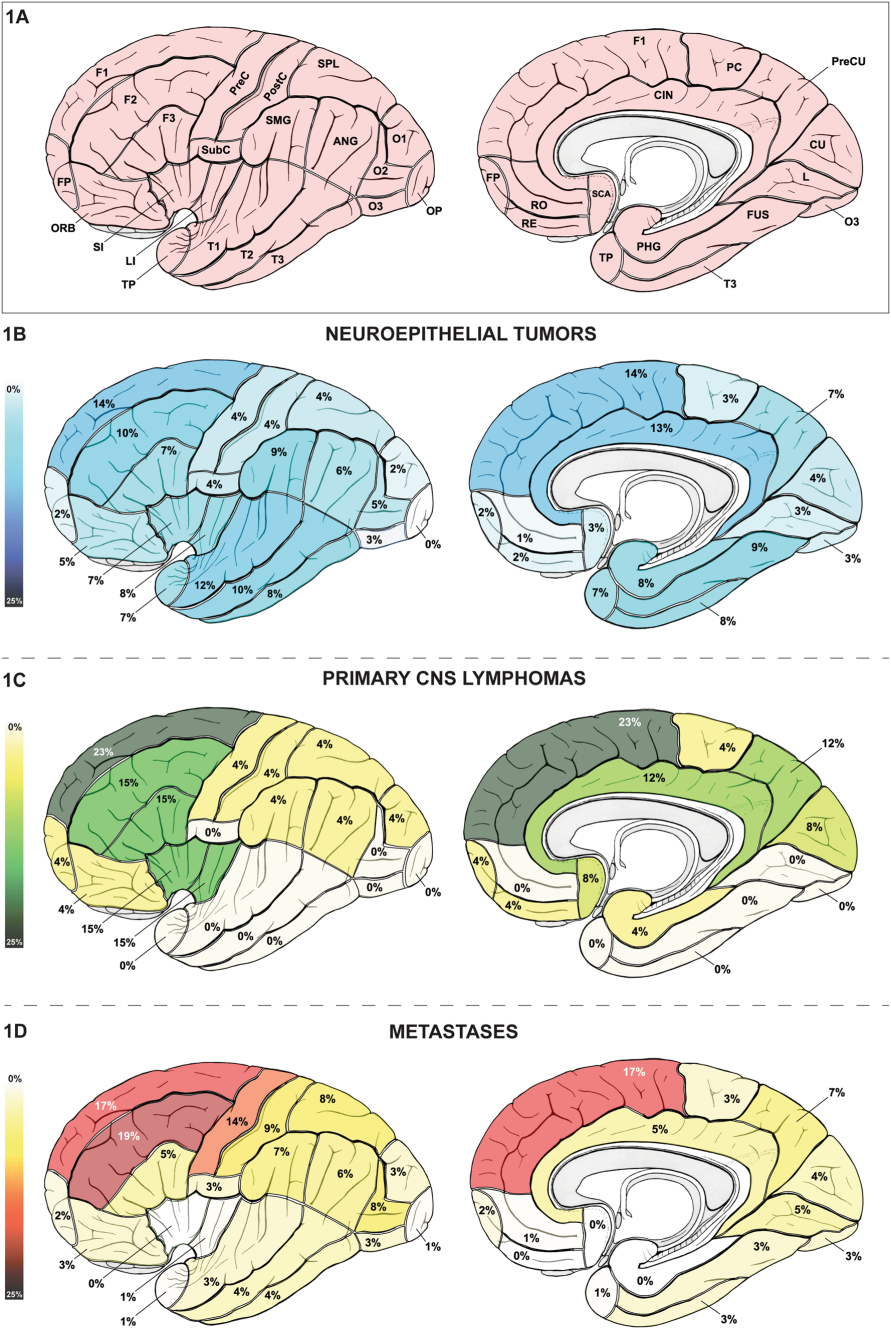
Surface topographic anatomy of supratentorial brain tumors: **A** lateral and medial view on the telencephalon and its gyral segments. **B** Gyral distribution pattern of neuroepithelial tumors. **C** Gyral distribution pattern of primary CNS (central nervous system) lymphomas. **D** Gyral distribution pattern of metastases. *ANG* angular gyrus, *CIN* cingulate gyrus, *CNS* central nervous system, *CU* cuneus, *F1* superior frontal gyrus, *F2* middle frontal gyrus, *F3* inferior frontal gyrus, *FP* frontal pole, *FUS* fusiform gyrus, *L* lingual gyrus, *LI* long insular gyri, *O1* superior occipital gyrus, *O2* middle occipital gyrus, *O3* inferior occipital gyrus, *OP* occipital pole, *ORB* orbital gyri, *PC* paracentral lobule, *PHG* parahippocampal gyrus, *PostC* postcentral gyrus, *PreC* precentral gyrus, *PreCU* precuneus, *RE* gyrus rectus, *RO* rostral gyrus, *SCA* subcallosal area, *SI* short insular gyri, *SMG* supramarginal gyrus, *SPL* superior parietal lobule, *SubC* subcentral gyrus, *T1* superior temporal gyrus, *T2* middle temporal gyrus, *T3* inferior temporal gyrus, *TP* temporal pole

**Fig. 2:**
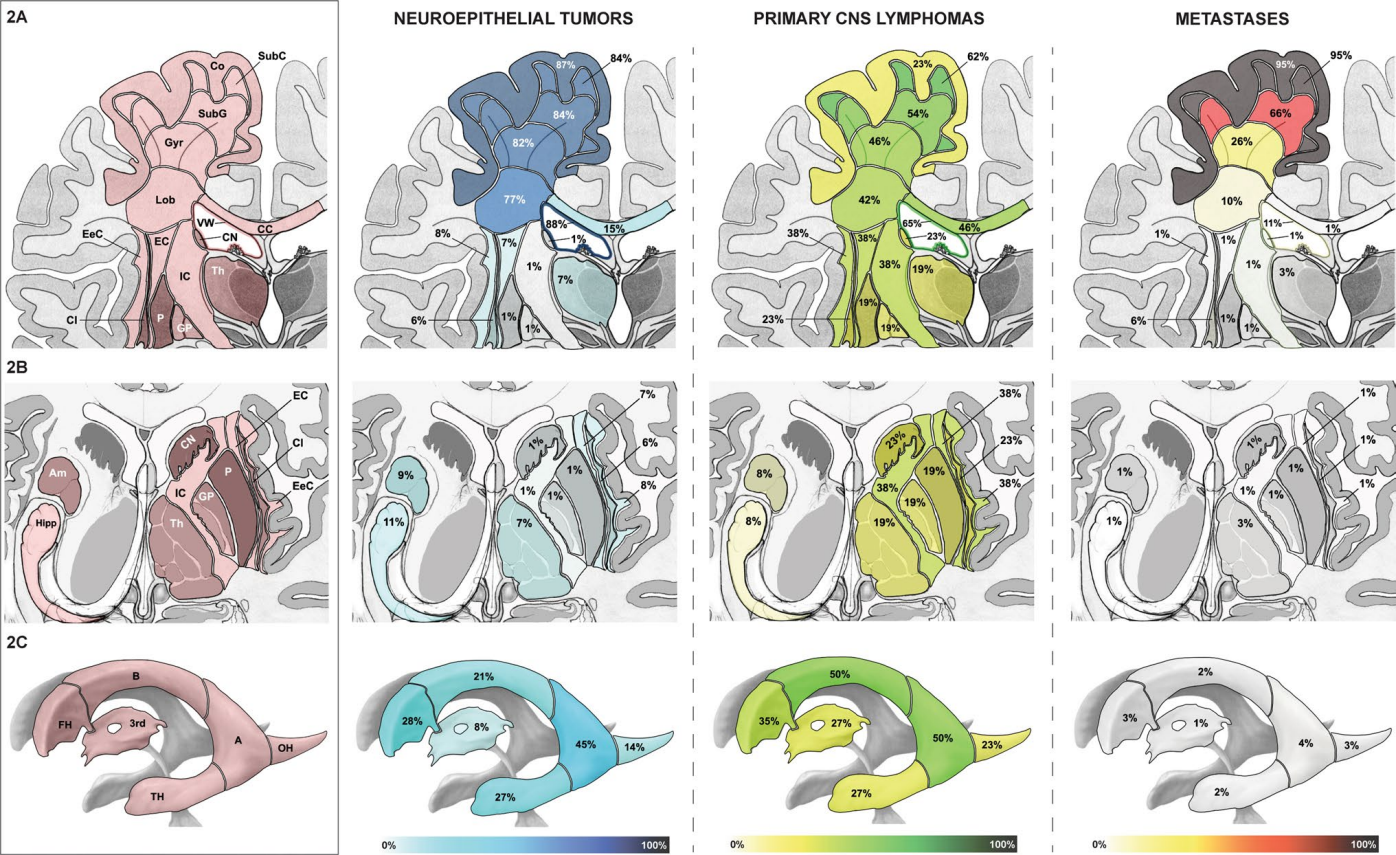
Deep topographic anatomy of supratentorial brain tumors. **A** Telencephalic cortex, white matter and deep gray matter: Coronal section through the telencephalon showing the cortex, white matter sectors (subcortical, subgyral, gyral, lobar), corpus callosum, ventricular wall, central white matter (extreme capsule, external capsule, internal capsule) and central gray matter (caudate nucleus, claustrum, putamen, globus pallidus, thalamus). The classification of the white matter into white matter sectors follows a structural dichotomous division. The prevalence of involvement of subcortical telencephalic structures in neuroepithelial tumors, primary CNS (central nervous system) lymphomas and metastases is shown. **B** Central telencephalic white and gray matter: Superior view on the telencephalic deep gray matter (caudate nucleus, claustrum, putamen, globus pallidus, thalamus, amygdala, hippocampus) and white matter (extreme capsule, external capsule, internal capsule) in a cross-section. The prevalence of involvement in neuroepithelial tumors, primary CNS (central nervous system) lymphomas and metastases is shown. **C** Supratentorial ventricular walls: Segments of the lateral ventricle (frontal horn, body, atrium, occipital horn, temporal horn) and 3rd ventricle from a left oblique view. The prevalence of contact of neuroepithelial tumors, primary CNS (central nervous system) lymphomas and metastases to the wall of the ventricular segments is shown. *3rd* third ventricle, *A* atrium, *Am* amygdala, *B* body of the lateral ventricle, *CC* corpus callosum, *Cl* claustrum, *CN* caudate nucleus, *CNS* central nervous system, *Co* Cortex, *EC* external capsule, *EeC* extreme capsule, *FH* frontal horn of the lateral ventricle, *Gyr* gyral white matter sector, *Hipp* hippocampus, *IC* internal capsule, *OH* occipital horn of the lateral ventricle, *P* putamen, *GP* globus pallidus, *SubC* subcortical white matter sector, *SubG* subgyral white matter sector, *TH* temporal horn of the lateral ventricle, *Th* thalamus, *VW* ventricular wall

**Fig. 3:**
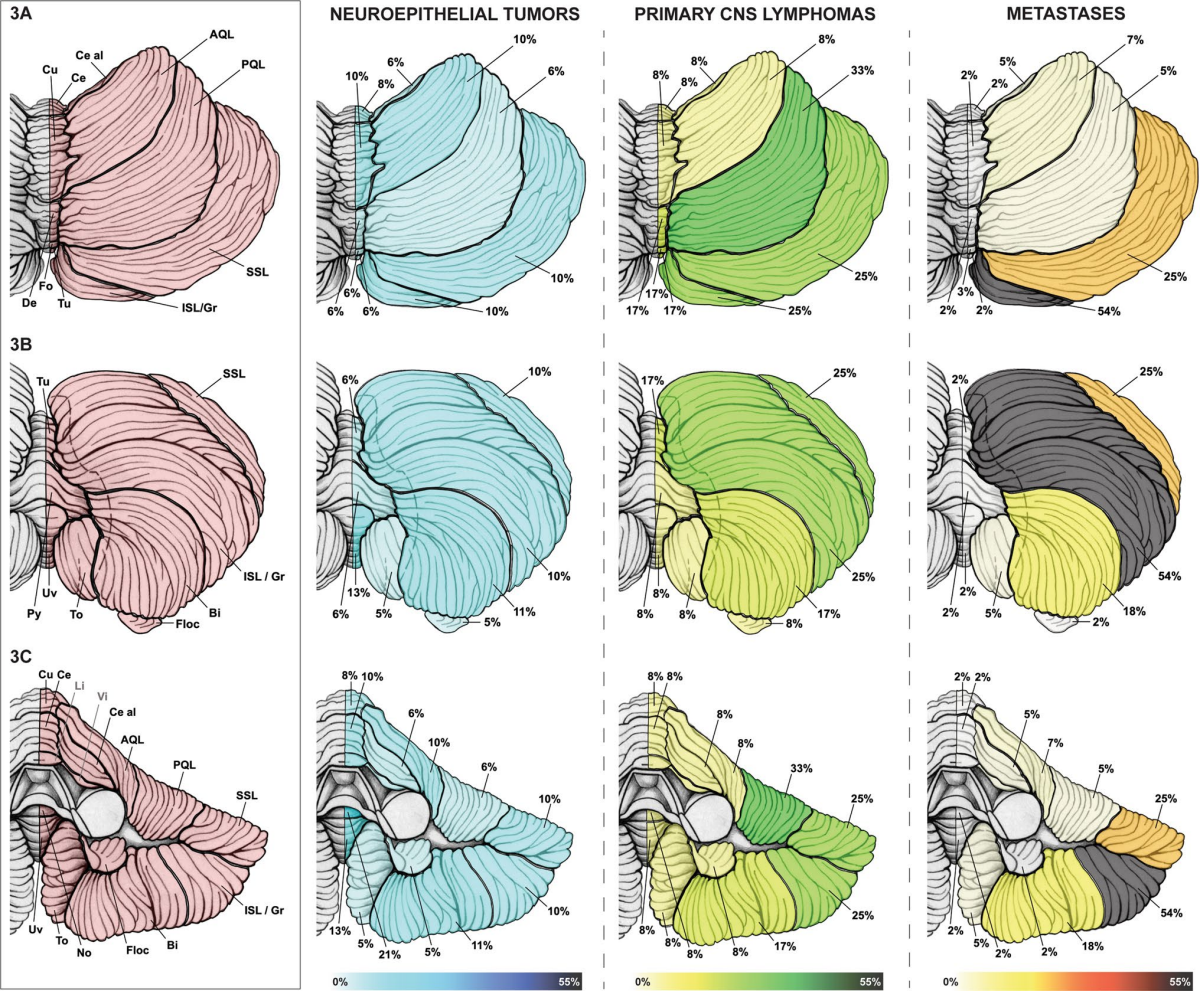
Surface topographic anatomy of cerebellar brain tumors: **A** superior, **B** inferior and **C** anterior view on the cerebellum with its vermian and corresponding hemispheric lobules. The cerebellar distribution of neuroepithelial tumors, primary CNS (central nervous system) lymphomas and metastases is shown. *AQL* anterior quadrangular lobule, *Bi* biventer/biventral lobule, *Ce* central lobule, *Ce al* ala lobuli centralis/wing of the central lobule, *CNS* central nervous system, *Cu* culmen, *De* declive, *Floc* flocculus, *Fo* folium, *ISL/Gr* inferior semilunar and gracile lobules—not separable on magnetic resonance imaging, *Li* lingual—not reliably separable on MRI, *No* nodule, *PQL* posterior quadrangular/simplex lobule, *Py* pyramid, *SSL* superior semilunar lobule, *To* tonsil, *Tu* tuber, *Uv* uvula, *Vin* vinculum—not reliably separable on MRI

**Fig. 4:**
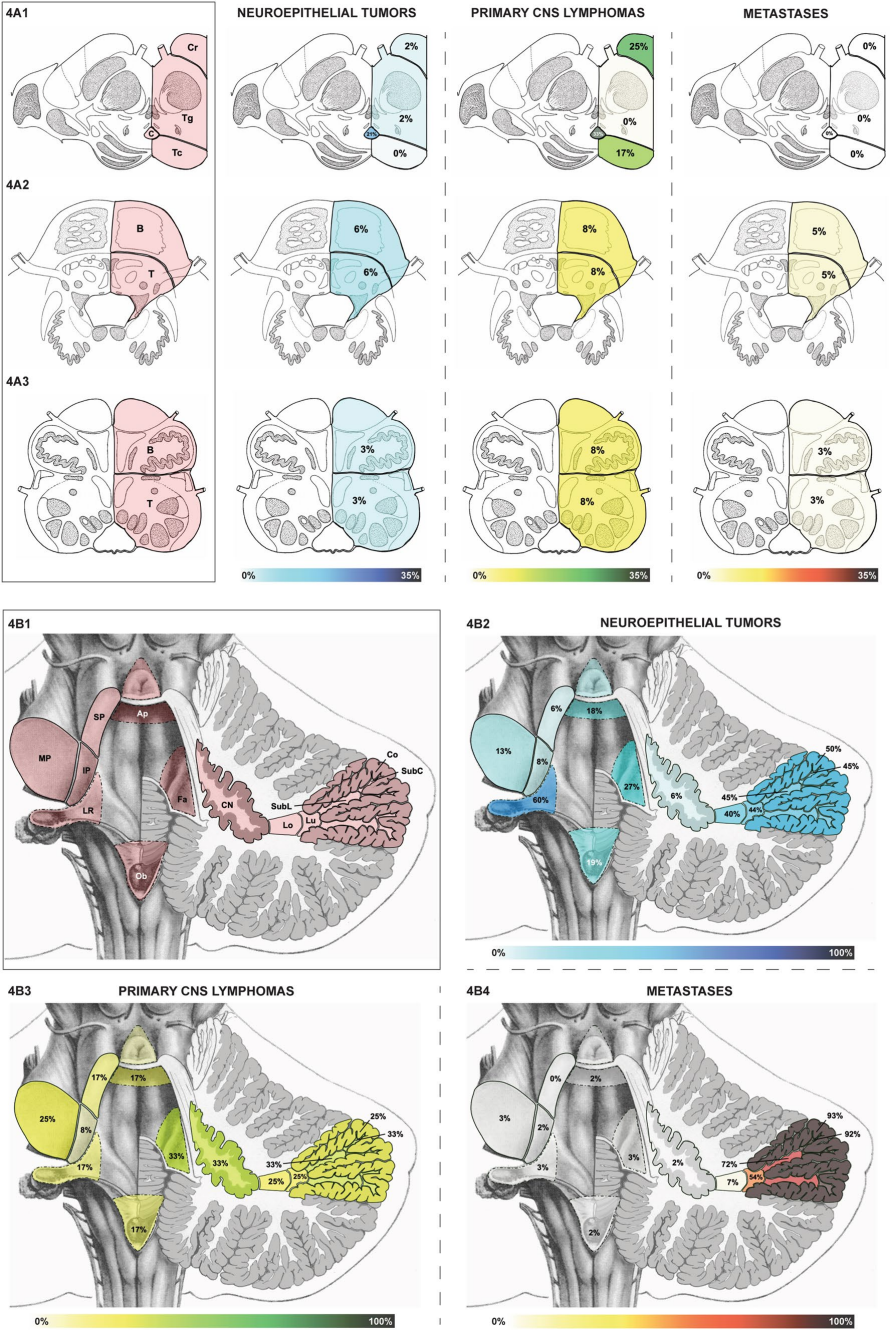
Brainstem and deep topographic anatomy of infratentorial brain tumors: **A**—brainstem: **A1** cross-section through the mesencephalon with its base, tegmentum, tectum and central aqueduct. **A2** Cross-section through the pons with its base and tegmentum. The frequency of involvement in neuroepithelial tumors, primary CNS (central nervous system) lymphomas and metastases is shown. **A3** Cross-section through the medulla oblongata with its base and tegmentum. The prevalence of involvement in neuroepithelial tumors, primary CNS (central nervous system) lymphomas and metastases is shown. **B**—Deep topographic anatomy of infratentorial brain tumors: **B1** Posterior view on the 4th ventricle (apex, lateral recesses, obex, fastigium), cerebellar peduncles (superior, middle, inferior) and subcortical cerebellar structures (white matter sector: subcortical, sublobular, sublobar, lobar; cerebellar nuclei). The prevalence of involvement in neuroepithelial tumours (**B2**), primary CNS (central nervous system) lymphomas (**B3**) and metastases (**B4**) is shown. *Ap* apex of the 4th ventricle, *C* central aqueduct, *CN* cerebellar nuclei, *CNS* central nervous system, *Co* cortex, *Fa* fastigium of the 4th ventricle, *IP* inferior cerebellar peduncle, *Lo* lobar white matter sector, *LR* lateral recess of the 4th ventricle, *Lu* lobular white matter sector, *MP* middle cerebellar peduncle, *Ob* obex of the 4th ventricle, *SP* superior cerebellar peduncle, *SubL* sublobular white matter sector, *SubC* subcortical white matter sector

Ethics board approval was obtained prior to data collection (KEK 2017–01120). All patients or their legal representatives gave their written informed consent. Demographic data gathering included gender, age at surgery and number of days between imaging and histopathological sampling. Histopathological analysis was performed by our Institute of Neuropathology. Histopathological diagnosis of primary brain tumors followed the 2007 WHO Classification [11], as the 2016 update [1] had not yet been released at time of histological evaluation. The MRI study closest to surgery and therefore histopathological sampling was used for image analysis to provide optimal correlation between imaging and histopathological information. Image analysis was performed by two authors (KA and CS) for each case independently and blinded for clinical and histopathological characteristics. In cases of disagreement, a consensus was found by involvement of the senior author (NK). Anatomical classification followed the Terminologia Anatomica [12] with the following modifications: On a lobar level, the concept of a prosencephalic central lobe was applied according to Yasargil et al. [13], comprising the precentral gyrus, postcentral gyrus, paracentral lobule and subcentral gyrus. Consequently, the mentioned structures were not considered to be part of the frontal or parietal lobe. As described previously [10], the extent of white matter involvement was assessed based on division of the hemispheric and cerebellar white matter (WM) into different segments (WM sectors) [13] based on structural dichotomia. The various anatomical structures are listed in Table 1 and illustrated in Figs. 1, 2, 3 and 4. Based on the investigator's experience, each anatomical structure was evaluated as either infiltrated or non-infiltrated by the tumor by visual determination combining all morphological sequences (T1, T1 with contrast, T2, FLAIR). Structures that were considered only displaced or edematous were not classified as affected.

**Table 1:** Topographic anatomy of supratentorial brain tumors: absolute and relative prevalence of invasion of supratentorial structures in neuroepithelial tumors, primary central nervous system lymphomas and metastases is shown

Cerebral structure	Prevalence of involvement in			p value			
	NT N = 374	PCNSL N = 26	M N = 178	Overall	NT vs. PCNSL	NT vs. M	PCNSL vs. M
Lobes							
* Unilobar*	*244 (72)*	*11 (61)*	*126 (74)*	*0.520*	*–*	*–*	*–*
* Multilobar*	*96 (28)*	*7 (39)*	*45 (26)*				
Frontal	107 (32)	13 (72)	75 (44)	< 0.001*	0.001*	0.011	0.023
Central	43 (13)	4 (22)	43 (25)	0.001*	0.487	0.001*	0.775
Parietal	77 (23)	7 (39)	48 (28)	0.154	*–*	*–*	*–*
Occipital	48 (14)	6 (33)	40 (24)	0.007*	0.054	0.025	0.358
Temporal	110 (32)	3 (17)	27 (16)	< 0.001*	0.321	< 0.001*	0.931
Insular	41 (12)	4 (22)	4 (2)	< 0.001*	0.207	0.001*	< 0.001*
Limbic	75 (22)	6 (33)	12 (7)	< 0.001*	0.269	< 0.001*	0.001*
Gyral segment							
* Unigyral*	*186 (55)*	*6 (33)*	*120 (70)*	< 0.001*	*0.074*	0.003*	0.003*
* Multigyral*	*153 (45)*	*12 (67)*	*51 (30)*				
Frontal pole	6 (2)	1 (4)	3 (2)	0.697	*–*	*–*	*–*
F1	51 (14)	6 (23)	31 (17)	0.268	*–*	*–*	*–*
F2	37 (10)	4 (15)	33 (19)	0.016	*–*	*–*	–
F3	27 (7)	4 (15)	9 (5)	0.142	–	–	–
Orbital	17 (5)	1 (4)	5 (3)	0.621	–	–	–
Rectus	8 (2)	1 (4)	0 (0)	0.104	–	–	–
Rostral	4 (1)	0 (0)	1 (1)	0.741	–	–	–
Subcallosal area	10 (3)	2 (8)	0 (0)	0.015	–	–	–
Precentral	14 (4)	1 (4)	25 (14)	< 0.001*	0.979	< 0.001*	0.290
Postcentral	13 (4)	1 (4)	16 (9)	0.023	–	–	–
Paracentral	10 (3)	1 (4)	5 (3)	0.939	–	–	–
Subcentral	14 (4)	0 (0)	5 (3)	0.534	–	–	–
Superior parietal lobule	16 (4)	1 (4)	14 (8)	0.204	–	–	–
Supramarginal	32 (9)	1 (4)	13 (7)	0.642	–	–	–
Angular	22 (6)	1 (4)	10 (6)	0.909	–	–	–
Precuneus	26 (7)	3 (12)	13 (7)	0.684	–	–	–
Cuneus	13 (4)	2 (8)	8 (4)	0.520	–	–	–
O1	9 (2)	1 (4)	6 (3)	0.766	–	–	–
O2	20 (5)	0 (0)	15 (8)	0.152	–	–	–
O3	8 (2)	0 (0)	6 (3)	0.484	–	–	–
Occipital pole	1 (0)	0 (0)	1 (1)	0.820	–	–	–
Lingual	13 (3)	0 (0)	9 (5)	0.387	–	–	–
Fusiform	32 (9)	0 (0)	6 (3)	0.027	–	–	–
T1	44 (12)	0 (0)	6 (3)	0.001*	0.128	0.004*	0.342
T2	37 (10)	0 (0)	7 (4)	0.015	–	–	–
T3	30 (8)	0 (0)	5 (3)	0.023	–	–	–
Temporal pole	28 (7)	0 (0)	2 (1)	0.003*	0.296	0.006*	0.587
Short insular	28 (7)	4 (15)	0 (0)	< 0.001*	0.151	< 0.001*	< 0.001*
Long insular	31 (8)	4 (15)	1 (1)	< 0.001*	0.216	0.001*	< 0.001*
Parahippocampal	30 (8)	1 (4)	0 (0)	< 0.001*	0.441	< 0.001*	0.017*
Cingulate	47 (13)	3 (12)	9 (5)	0.024	–	–	–
Cortical involvement	325 (87)	6 (23)	169 (95)	< 0.001*	< 0.001*	0.004*	< 0.001*
WM (peripheral)	330 (88)	17 (65)	170 (96)	< 0.001*	0.002*	0.006*	< 0.001*
Subcortical sector	313 (84)	16 (62)	169 (95)	< 0.001*	0.004*	< 0.001*	< 0.001*
Subgyral sector	314 (84)	14 (54)	118 (66)	< 0.001*	< 0.001*	< 0.001*	0.215
Gyral sector	305 (82)	12 (46)	47 (26)	< 0.001*	< 0.001*	< 0.001*	0.038
Lobar sector	288 (77)	11 (42)	18 (10)	< 0.001*	< 0.001*	< 0.001*	< 0.001*
Corpus callosum	55 (15)	12 (46)	2 (1)	< 0.001*	< 0.001*	< 0.001*	< 0.001*
Capsule	31 (8)	11 (42)	3 (2)	< 0.001*	< 0.001*	0.003	< 0.001*
Internal capsule	2 (1)	10 (38)	2 (1)	< 0.001*	< 0.001*	0.446	< 0.001*
External capsule	26 (7)	10 (38)	2 (1)	< 0.001*	< 0.001*	0.004*	< 0.001*
Extreme capsule	30 (8)	10 (38)	2 (1)	< 0.001*	< 0.001*	0.001*	< 0.001*
Deep gray matter	82 (22)	10 (38)	10 (6)	< 0.001*	0.053	< 0.001*	< 0.001*
Caudate nucleus	4 (1)	6 (23)	2 (1)	< 0.001*	< 0.001*	0.954	< 0.001*
Putamen	4 (1)	5 (19)	2 (1)	< 0.001*	< 0.001*	0.954	< 0.001*
Globus pallidus	5 (1)	5 (19)	1 (1)	< 0.001*	< 0.001*	0.412	< 0.001*
Claustrum	23 (6)	6 (23)	2 (1)	< 0.001*	0.003*	0.008*	< 0.001*
Hypothalamus	17 (5)	5 (19)	0 (0)	< 0.001*	0.004*	0.004*	< 0.001*
Thalamus	28 (7)	5 (19)	5 (3)	0.003*	0.061	0.061	0.001*
Amygdala	35 (9)	2 (8)	1 (1)	< 0.001*	0.777	< 0.001*	0.010*
Hippocampus	43 (11)	2 (8)	1 (1)	< 0.001*	0.553	< 0.001*	0.010*
Ventricular wall	330 (88)	17 (65)	19 (11)	< 0.001*	0.001*	< 0.001*	< 0.001*
* Unisegmental*	*209 (63)*	*3 (18)*	*16 (80)*	< 0.001*	< 0.001*	*0.123*	< 0.001*
* Multisegmental*	*123 (37)*	*14 (82)*	*4 (20)*				
Frontal horn	106 (28)	9 (35)	5 (3)	< 0.001*	0.494	< 0.001*	< 0.001*
Body	80 (21)	13 (50)	4 (2)	< 0.001*	0.001*	< 0.001*	< 0.001*
Atrium	168 (45)	13 (50)	7 (4)	< 0.001*	0.615	< 0.001*	< 0.001*
Occipital horn	52 (14)	6 (23)	6 (3)	< 0.001*	0.199	< 0.001*	< 0.001*
Temporal horn	101 (27)	7 (27)	4 (2)	< 0.001*	0.933	< 0.001*	< 0.001*
3rd ventricle	30 (8)	7 (27)	2 (1)	< 0.001*	0.002*	0.002*	< 0.001*

Significant p-values are marked with an asterisk

N = 578

*NT* neuroepithelial tumor, *PCNSL* primary central nervous system lymphoma, *M* metastases, *F1* superior frontal gyrus, *F2* middle frontal gyrus, *F3* inferior frontal gyrus, *O1* superior occipital gyrus, *O2* middle occipital gyrus, *O3* inferior occipital gyrus, *T1* superior temporal gyrus, *T2* middle temporal gyrus, *T3* inferior temporal gyrus, *WM* white matter

### Statistical analysis

Continuous variables are reported as means and standard deviations, categorical data as numbers and percentages. For control of test multiplicity, we applied a closed testing procedure. The three groups were compared using Pearson’s χ^2^ tests for categorical data and one-way analysis of variance (ANOVA) for continuous data. A *p* < 0.01 on a two-tailed test was considered significant. Upon significance of the omnibus test, pairwise comparisons were carried out. Here, *p* values were adjusted using the Holm-Bonferroni method. All analyses were carried out in R version 3.5.1 (The R Foundation for Statistical Computing, Vienna, Austria).

## Results

Of 690 patients with intraparenchymal neoplasms 42 (6%) were excluded due to inconclusive histopathological results (n = 21, 3.0%), incomplete MRI data (n = 19, 2.8%) or previous cranial surgery (n = 2, 0.3%) (Supplementary Table S1). Of the 648 patients included, 419 (65%) were diagnosed with a NT, 28 (5%) with a PCNSL and 201 (31%) with metastases. Epidemiologic cohort data and details on histopathological results are provided in Supplementary Tables S1 and S2, respectively.

The relationship of NT, PCNSL and metastases to the tentorium and the midline are detailed in Supplementary Table S3. While all three entities were more likely to occur above the tentorium, NT showed a disproportionately increased propensity to supratentorial structures (85%), compared to PCNSL (57%) and metastases (70%) (p < 0.001). PCNSL (36%) and metastases (19%) were, in comparison to NT (4%), more likely to involve both supra- and infratentorial spaces (p < 0.001). No right-left-differences were found in any of the tumor types, but PCNSL were significantly more frequently bilateral (64%) than NT (23%) or metastases (30%) (p < 0.001).

### Supratentorial structures

Table 1 and Figs. 1 and 2 characterize the prevalence of supratentorial structural involvement.

#### Lobes and gyral segments

Absolute and relative frequencies of involvement of the cerebral lobes and gyri are given in Table 1 and illustrated in Fig. 1. NT, PCNSL and metastases significantly differed in lobar and gyral topographic anatomy. Cerebral parenchymal involvement remained unigyral in 55% of NT, 33% of PCNSL and 70% of metastases, respectively (p < 0.001).

#### Cortex, white matter sectors, corpus callosum, capsule and deep gray matter

As shown in Table 1 and Fig. 2A, metastases, NT and PCNSL differed in their pattern of cortex and white matter sector involvement. Metastases affected the cortex and subcortical WM in 95% of cases with a marked decrease towards more central layers, with only 10% affecting the lobar WM sector. While the cortex was involved in 87% of NT, only a discrete decrease in frequency towards more central sectors was observed. PCNSL affected the cortex in only 23%, whereas white matter sectors were more frequently involved (42–62%). The corpus callosum was affected in 15% of NT, 46% of PCNSL and 1% of metastases (p < 0.001). The internal capsule was involved in only 1% of NT and metastases, but in 38% of PCNSL (p < 0.001). Deep gray matter structures were affected by 6% of metastases, 22% of NT and 38% of PCNSL (p < 0.001) (Table 1, Fig. 2A and B).

#### Ventricular wall

NT contacted the ventricle in 88% of cases, PCNSL in 65% and metastases in 11% (p < 0.001) (Table 1 and Fig. 2A). NT, PCNSL and metastases differed in their topographic-anatomical pattern of contact to the segments of the lateral (frontal horn, body, atrium, occipital and temporal horn) and 3rd ventricle, as is shown in Table 1 and Fig. 2C.

### Infratentorial structures

Supplementary Table S4 and Figs. 3 and 4 characterize the prevalence of infratentorial structural involvement.

#### Brainstem—mesencephalon, pons and medulla oblongata

In the brainstem, differences in the topographic anatomy were mainly found in the mesencephalon (Supplementary Table S4 and Fig. 4A1). It was frequently involved with infratentorial PCNSL (67%), while rarely with NT (24%) and never with metastases. Mesencephalic NT showed a clear propensity to the aqueductal region (13/15, 87%). While the crus cerebri was rarely affected by mesencephalic NT (7%, 1/15), it was commonly involved in mesencephalic PCNSL (3/8, 38%).

#### Cerebellum and 4th ventricle

As shown in Supplementary Table S4 and Fig. 3, NT, PCNSL and metastases differed in their cerebellar topographic anatomy. 93% of infratentorial metastases were found in the cerebellar hemispheres, while only 3% were located in the vermis. The posterior cerebellar lobe harbored 66% of all infratentorial metastases and the most commonly affected lobule was the inferior semilunar/gracile (54%). NT and PCNSL showed no significant difference between vermian and hemispheric involvement.

As shown in Supplementary Table S4 and Fig. 4B, also in the cerebellum a significant difference regarding the pattern of cortex and white matter involvement was found between the tumor entities. The cerebellar cortex was affected by 91% (28/34) of cerebellar NT, 60% (3/5) of PCNSL and 98% (57/58) of cerebellar metastases. Whereas a marked decrease in frequency of involvement towards deeper white matter sectors was seen in cerebellar metastases (Fig. 4B4), NT (Fig. 4B2) showed a more uniform involvement of the sectors along the cortico-ventricular axis. PCNSL (Fig. 4B3) more commonly involved the cerebellar white matter than the cortex and showed the highest frequency of cerebellar peduncular invasion.

A contact to the 4th ventricle was found in 72% of infratentorial and 100% (34/34) of cerebellar NT (Fig. 4B2), while PCNSL (Fig. 4B3) and metastases (Fig. 4B4) showed significantly lower contact rates. Contact to the lateral recess of the 4th ventricle was overrepresented in NT (80% of cerebellar and 60% of infratentorial NT, p < 0.001).

## Discussion

This study provides a comprehensive anatomical description of the topography of NT, PCNSL and metastases intended to serve as a topographic reference for clinicians and neuroscientists. The identified distinct anatomical patterns provide evidence for a specific interaction between tumor and anatomical structures, following a pathoclitic concept.

### Topographic anatomy of neuroepithelial tumors

**Fig. 5:**
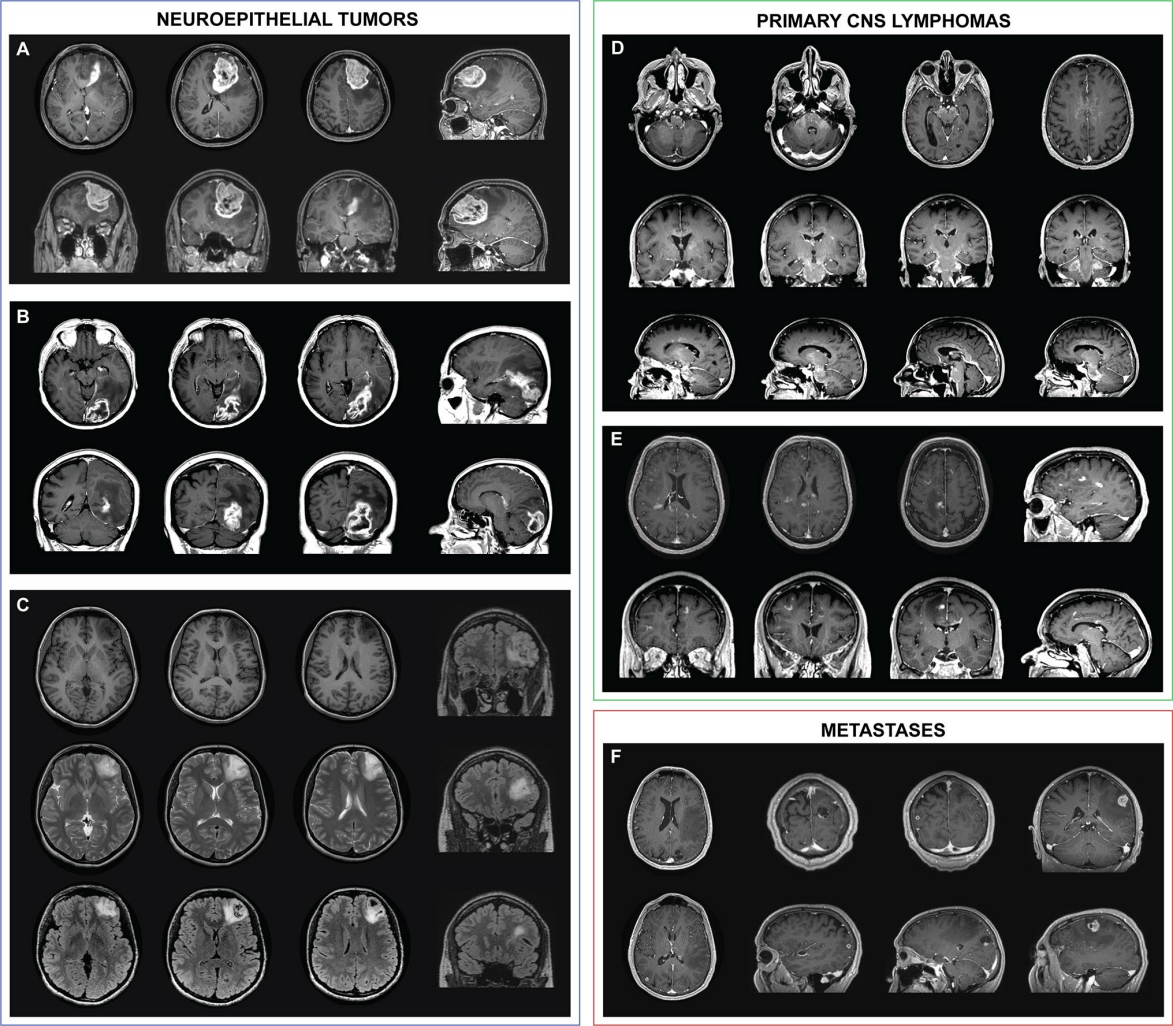
Illustrative magnetic resonance imaging based topographic anatomical tumor behavior: **A**—glioblastoma: example of a patient with glioblastoma of the left superior frontal gyrus. Despite the extended character of the tumor, a segmental topographic-anatomical behavior with displacement of the adjacent gyri and a tail-like relationship to the wall of the frontal horn is observed. **B**—Glioblastoma: another case of a patient with glioblastoma of the left occipital pole and superior occipital gyrus. Despite the extended character of the tumor, the spatial character along the ventriculo-cortical radial unit is preserved. **C**—WHO grade II astrocytoma: example of a patient with grade II astrocytoma of the left medial frontal gyrus. The segmental topographic-anatomical behavior of the tumor with tail-like relationship to the wall of the left frontal horn becomes evident here as well. **D**—Primary CNS lymphoma: this example of a patient with primary CNS lymphoma illustrates the topographic-anatomical pattern of these tumors along the projection and commissural white matter fibers. The tumor can be followed from the internal capsule, through the cerebral peduncles via the pons to the ventral medulla oblongata and the cerebellar peduncles. In addition, an infiltration along the corpus callosum can be seen. **E**—Primary CNS lymphoma: another example of a patient with primary CNS lymphoma, which demonstrates the tumor distribution along the short association fibers. **F**—Multiple metastases: a patient with multiple intracranial metastases due to an adenocarcinoma of the lung. This example illustrates both the cortico-subcortical character and the topographic-anatomical preference for watershed areas of metastases. *CNS* central nervous system

The widespread belief of gliomas extending along WM tracts [14] cannot be supported by our topographic-anatomical findings, instead implying an expansion along scaffolds with a ventriculo-cortical direction. First, a spread along association fibers is highly unlikely as NT remained unigyral in 55% of cases, whereas cortical and ventricular contact was observed in 87% and 88%, respectively. Second, invasion along commissural fibers seems implausible, as the corpus callosum was involved in only 15% of NT, which is markedly less than in PCNSL (46%) and significantly rarer than NT involvement of structures anatomically adjacent to the corpus callosum such as the ventricle (88%) or the lobar (77%) or gyral (82%) WM sector. Third, evidence against a spread along projection fibers is provided by the fact that an invasion of the internal capsule was seen in only 1% of NT, again significantly less than with PCNSL (38%) and despite high frequency of invasion of adjacent structures [ventricle (88%), lobar white matter sector (77%), gyral white matter sector (82%)]. Instead, NT showed a radial orientation with involvement of the entire ventriculo-cortical distance, while remaining limited in their transverse extension (supratentorial: cortex 87%, ventricle 88%, unigyral 55%; cerebellar: cortex 91%, ventricle 100%, unilobar 74%) (Fig. 5A–C). This implies an orientation along scaffolds with a ventriculo-cortical axis. The trajectory of white matter tracts does not correspond to this axis. The perivascular spaces [15], that form part of the transcerebral CSF pathway, and radial glia-like cells [16, 17], representing the ontogenetic neuroglial migration pathway, however, are potential scaffolds showing a corresponding orientation.

The preference of NT for certain lobes, gyri and ventricular segments indicates a non-uniform distribution of origin cells along the neuraxis. Subventricular astrocyte-like neural stem cells are considered a likely origin [18] and are known to be non-uniformly distributed within the periventricular zone [19]. Both, a subventricular origin and an orientation along a defined ventriculo-cortical axis parallel developmental processes during ontogenesis [20]. The analogy between gliomagenesis and ontogenesis is supported by molecular evidence, showing the stages of gliomagenesis to retrogradely parallel the stages of ontogenesis [21]. NT cells might therefore—by re-expression of previously silenced migration genes—use similar migration scaffolds as during ontogenesis, i.e. radial glia-like cells. It is therefore conceivable that subventricular stem-like cells, after acquisition of such mutations, behave analogously to their ontogenetic coding within their ventriculo-cortical radial unit. A periventricular tumor origin also has to be considered infratentorially. Mesencephalic NT were found to be located around the aqueduct in 87% of cases and every cerebellar NT contacted the wall of the 4th ventricle. The high contact rate of NT to the lateral recess of the 4th ventricle (80% of those with contact to the 4th ventricle, 60% of infratentorial NT) indicates that this region may be of importance with regard to infratentorial NT origin. Little is known about the infratentorial distribution of stem-like cells and the possibility of periventricular origin of NT has never been investigated.

### Topographic anatomy of primary CNS lymphomas

The topographic anatomy of PCNSL is compatible with an orientation along white matter tracts, along association, commissural and projection fibers (Fig. 5D and E). First, in contrast to NT and metastases, PCNSL were often multigyral (67%) or even multilobar (39%). Whereas the cortex was involved in only 23% of cases, the underlying subcortical WM was affected in 62% with minor decrease towards the lobar WM sector (42%). An extension along short association fibers explains the cortical sparing and wrapping around sulci towards the adjacent gyri [22]. Long association fibers, localized in deeper WM sectors, may serve as a scaffold for a more distant spread, such as to other lobes. Second, PCNSL were shown to be often bilateral (64%) with involvement of the corpus callosum in 46% of cases. This makes an extension to the contralateral hemisphere as frequent as to the anatomically adjacent lobar WM sector (42%), indicating an ability of PCNSL to spread along the commissural system. Third, PCNSL invasion along projection fibers may explain the involvement of the internal capsule with similar frequency to the lobar WM sector (38% versus 42%), whereas the adjacent gray matter structures (striatum, thalamus, hypothalamus) were involved less frequently (19–23%). Infratentorial projection fiber systems, such as the crus cerebri (25%) or the cerebellar peduncles (33%) showed also more frequent involvement than in NT or metastases.

### Topographic anatomy of brain metastases

Metastases showed a propensity towards the cortex and subcortical WM consistent with the hypothesis of metastases preferentially lodging at the gray-white matter junction [23] (Fig. 5F). Supratentorially, these structures were involved in 96% and 95% of cases, respectively, with a marked decrease towards the lobar WM sector (10%). In the cerebellum, the cortex and the subcortical WM sector were affected in 98% and 97%, diminishing markedly to the lobar WM sector (7%). The low prevalence in central supratentorial structures, brainstem and central cerebellar structures indicates a low probability of metastatic seeding via perforators.

The identified topographic-anatomical patterns in metastases correspond to the large arteries’ watershed areas, both supra- and infratentorially (Fig. 5F). Gyri virtually exclusively supplied by the anterior, middle or posterior cerebral artery, were affected less by metastases (Supplementary Table S5). In contrast, the gyri with a high prevalence of metastases were those in border areas of these vessels (Supplementary Table S5). Infratentorially, the cerebellar hemisphere was significantly overrepresented, harboring 93% of all posterior fossa metastases. They were most commonly found within the superior semilunar (25%) or inferior semilunar/gracile (54%) and the biventer (18%) lobules, again consistent with watershed areas. Lower frequencies were seen in those structures generally supplied by a single cerebellar artery (Supplementary Table S5). Therefore, our topographic-anatomical data support the theory of metastases seeding to terminal vessel branches [23].

### Limitations

An important limitation of this study remains observer-dependency of image analysis. To minimize this bias, radiological analysis was performed by multiple raters, blinded for clinical and histopathological information and a strictly defined topographic-anatomical classification with a binary stratification (affected vs. non-affected) was used. Secondly, areas considered as edematous were not included in the analysis. This represents a limitation in that such areas may also be infiltrated by tumor cells. However, no differentiation between edema with and without tumor cells can be made on the basis of imaging alone. To ensure a high level of interrater reliability, these areas were deliberately excluded. Third, the prevalence of tumor involvement of individual anatomical structures was not normalized to their volume. However, the comparisons between the different tumor entities are non-affected by this. Fourth, the low case number in certain anatomical and histological groups limits statistical significance. To achieve optimal topographic accuracy, the brain was partitioned into many anatomical regions, leading to small sample sizes in some regions. Furthermore, the number of PCNSL was limited, resulting in reduced statistical power for comparisons with PCNSL. And fifth, a large number of statistical tests were required due to the detailed anatomical partitioning of brain regions. We therefore chose to reduce the level of significance to *p* < 0.01 in an effort to limit Type I errors and applied a closed testing procedure to control the family-wise error rate. It should also be noted that a temporal component is missing in our data and therefore no direct statement on the tumor growth behavior can be given. Our topographic-anatomical analyses only provide indirect conclusions in this respect based on the observed patterns and by comparison between NT, PCNSL and metastases. Finally, in the context of the current WHO classification with emphasis on histological and molecular subtypes, a holistic classification in NT might appear counterintuitive. However, the aim of this study was to investigate the interaction of tumors with cerebral anatomy with regard to their ontogenetic background. Since all NT entities share a common developmental origin and ontogenetic neuroepithelial coding, they were analyzed collectively. For further analyses comparing the topographic-anatomical patterns of different neuroepithelial subentities or metastases of various origin, larger sample sizes are required to ensure sufficient statistical power for such detailed anatomical classification.

### Clinical relevance

Whereas the 2016 WHO classification of CNS tumors represents an important step towards a multimodal-integrated classification system [1], macroscopic topographic-anatomical features have—in contrast to microscopic and molecular characteristics—not been implemented, although they might represent a valuable complement. First, in contrast to histological and molecular features which require biological material in order to be assessed, topographic-anatomical patterns are based on imaging studies which are available early in the preoperative diagnostic process and therefore have the potential to influence treatment strategy. Secondly, since it is based on structural criteria only, the topographic-anatomical classification is independent of advanced imaging techniques, which are usually unavailable in the initial clinical setting. Third, topographic-anatomical features have the significant advantage of not being influenced by a possible sampling error due to intratumor heterogeneity [24]. Appreciation of the topographic-anatomical pattern underlying a brain tumor may lead to anatomically guided surgery and radiotherapy. Whenever functionally justifiable, a surgeon should aim at maximal cytoreduction with NT [25]. An anatomically tailored supratotal resection, encompassing the whole VCRU, including the potential periventricular stem-like cells, may improve clinical outcome. Radiotherapy may follow the same anatomical principles with irradiation of the whole VCRU. An anatomically tailored therapy is further supported by the fact that irradiation of the ipsilateral subventricular zone has been shown to significantly improve survival in glioblastoma patients [26].

## Conclusions

This study provides a comprehensive anatomical description of the topography of NT, PCNSL and metastases intended to serve as a topographic reference for clinicians and neuroscientists. The identified distinct anatomical patterns provide evidence for a specific interaction between tumor and anatomical structures, following a pathoclitic concept. Understanding differences in their anatomical behavior has the potential to improve our pathophysiological understanding and to tailor therapy of brain tumors.

## Electronic supplementary material

Below is the link to the electronic supplementary material.

Supplementary file1 (DOCX 16 kb)—Online Resource 1, Methods:The supplemental Digital Content expands on the technical magnetic resonance imaging (MRI)data.

Supplementary file2 (DOCX 39 kb)

Supplementary file3 (R 0 kb)—Statistical (R) code

Supplementary file4 (PDF 117 kb)

## Data Availability

Additional data is made available in the supplementary methods and supplementary results online. The statistical software (R) code used is made available in the supplementary methods online.
