# Supplementary material for: Distinct topographic-anatomical patterns in primary and secondary brain tumors and their therapeutic potential
Source: J Neurooncol. 2020 Jul 8;149(1):73–85. doi: 10.1007/s11060-020-03574-w (PMC7452943; doi:10.1007/s11060-020-03574-w)
Supplement: Supplementary file 1 — Supplementary file1 (DOCX 16 kb)—Online Resource 1, Methods:The supplemental Digital Content expands on the technical magnetic resonance imaging (MRI)data. [file 11060_2020_3574_MOESM1_ESM.docx]

**Distinct topographic-anatomical patterns in primary and secondary brain tumors and their therapeutic potential**

***Journal of Neuro-Oncology***

Kevin Akeret^1^* MD; Victor E. Staartjes^1^*, BMed; Flavio Vasella^1^, MD; Carlo Serra^1^, MD; Jorn Fierstra^1^, MD, PhD; Marian Christoph Neidert^1^, MD; Luca Regli^1^, MD; Niklaus Krayenbühl^1,2^, MD.

^1^Department of Neurosurgery, Clinical Neuroscience Center, University Hospital Zurich, University of Zurich, Zurich, Switzerland.

^2^Division of Pediatric Neurosurgery, University Children's Hospital, Zurich, Switzerland.

**Contributed equally*

*Corresponding author:*

Kevin Akeret

Department of Neurosurgery

University Hospital Zurich

Frauenklinikstrasse 10

CH-8091 Zurich, Switzerland

Telephone: +4144 255 11 11

E-mail: kevin.akeret@gmx.ch

**Supplementary Methods**

**Technical Magnetic Resonance Imaging (MRI) data**

3 Tesla Skyra VD13 (Siemens, Erlangen, Germany), 32-channel head coil; high resolution 3D T1-weighted anatomical sequence planned on the ACPC line plus 20° on a sagittal image with voxel size 0.8 × 0.8 ×1.0 mm3 with a Field of View 230 × 230 mm2 and resolution of 288 × 288, 176 slices per slab with a thickness of 1 mm, TR/TE 2200/5.14 ms, TI 900 ms, flip angle 8°; FLAIR images acquired with same orientation as T1-weighted images, voxel size 0.9 × 0.9 ×1.0 mm3 with a Field of View 230 × 230 mm2 and resolution of 256× 256, 176 slices per slab with a thickness of 1 mm, TR/TE 4000/387 ms, TI 1800 ms.
