# Supplementary material for: Distinct topographic-anatomical patterns in primary and secondary brain tumors and their therapeutic potential
Source: J Neurooncol. 2020 Jul 8;149(1):73–85. doi: 10.1007/s11060-020-03574-w (PMC7452943; doi:10.1007/s11060-020-03574-w)
Supplement: Supplementary file 2 — Supplementary file2 (DOCX 39 kb) [file 11060_2020_3574_MOESM2_ESM.docx]

**Distinct topographic-anatomical patterns in primary and secondary brain tumors and their therapeutic potential**

***Journal of Neuro-Oncology***

Kevin Akeret^1^* MD; Victor E. Staartjes^1^*, BMed; Flavio Vasella^1^, MD; Carlo Serra^1^, MD; Jorn Fierstra^1^, MD, PhD; Marian Christoph Neidert^1^, MD; Luca Regli^1^, MD; Niklaus Krayenbühl^1,2^, MD.

^1^Department of Neurosurgery, Clinical Neuroscience Center, University Hospital Zurich, University of Zurich, Zurich, Switzerland.

^2^Division of Pediatric Neurosurgery, University Children's Hospital, Zurich, Switzerland.

**Contributed equally*

*Corresponding author:*

Kevin Akeret

Department of Neurosurgery

University Hospital Zurich

Frauenklinikstrasse 10

CH-8091 Zurich, Switzerland

Telephone: +4144 255 11 11

E-mail: kevin.akeret@gmx.ch

**Supplementary Tables**

**Supplementary Table S1 – Screening, Demographic and Technical Information:** The number of patients screened, included and excluded are shown, as well as the exclusion criteria. Demographic (gender and age) data refer only to the included subjects. *Abbreviations:* N°: number; NT: neuroepithelial tumor; PCNSL: primary central nervous system lymphoma; M: metastases; MRI: magnetic resonance imaging.

| **Category** | **N°** | | | | p value |
| --- | --- | --- | --- | --- | --- |
|  | **Overall** | **NT** | **PCNSL** | **M** |  |
| **Screened patients** | **690 (100)** | **431 (100)** | **30 (100)** | **208 (100)** |  |
| Included patients | **648 (94)** | **419 (97)** | **28 (93)** | **201 (97)** |  |
| Insufficient MRI data | **19** | **12** | **2** | **5** |  |
| Previous cranial surgery | **2** | **0** | **0** | **2** |  |
| Uncertain histology | **21** | **-** | **-** | **-** |  |
| **Male Gender** | **372 (57)** | **256 (61)** | **14 (50)** | **102 (51)** | **0.037** |
| **Age at Surgery** | **53.5 ± 19.4** | **50.0 ± 21.2** | **58.5 ± 18.3** | **60.1 ± 12.5** | **< 0.001*** |
| **Days between most recent imaging and surgery/histopathology** | **5.3 ± 12.7** | **4.7 ± 7.0** | **3.8 ± 2.9** | **6.7 ± 20.3** | **0.078** |

**Supplementary Table S2 – Tumor Characteristics:** Histopathological details of the included subjects are shown based upon the 2007 WHO Classification^18^. *Abbreviations:* N°: number; 1°: primary; 2°: secondary; AT/RT: atypical teratoid/rhabdoid tumor; PXA: pleomorphic xanthastrocytoma; DNET: dysembryoplastic neuroepithelial tumor; CML: chronic myeloid leukemia.

| **Category** | **N° of patients**  **N = 648** |
| --- | --- |
| **Primary vs secondary** |  |
| 1° | 447 (69) |
| 2° | 201 (31) |
| **Neuroepithelial Tumors** | **419 (65)** |
| ***Grade IV*** | ***263 (63)*** |
| Glioblastoma | 250 (39) |
| Medulloblastoma | 12 (2) |
| AT/RT | 1 (0) |
| ***Grade III*** | ***75 (18)*** |
| Astrocytoma | 45 (7) |
| Oligoastrocytoma | 11 (2) |
| Oligodendroglioma | 9 (2) |
| Anaplastic PXA | 1 (0) |
| Ganglioglioma | 9 (2) |
| Ependymoma | 9 (2) |
| ***Grade II*** | ***40 (10)*** |
| Astrocytoma | 11 (2) |
| Oligoastrocytoma | 7 (1) |
| Oligodendroglioma | 7 (1) |
| Pilomyxoid Astrocytoma | 3 (0) |
| Ganglioglioma | 1 (0) |
| Central Neurocytoma | 3 (0) |
| Ependymoma | 6 (1) |
| Plexus Papilloma | 2 (0) |
| ***Grade I*** | ***40 (10)*** |
| Pilocytic Astrocytoma | 21 (3) |
| Ganglioglioma | 1 (0) |
| DNET | 2 (0) |
| Subependymal Giant Cell Astrocytoma | 1 (0) |
| Subependymoma | 4 (1) |
| Plexus Papilloma | 2 (0) |
| **Primary Central Nervous System Lymphoma** | **28 (5)** |
| **Metastases** | **201 (31)** |
| Adenocarcinoma (unknown primary) | 2 (0) |
| Bronchus | 95 (15) |
| Pleural Mesothelioma | 1 (0) |
| Mamma | 20 (3) |
| Oral | 3 (0) |
| Tonsillar Squamous Cell | 1 (0) |
| Esophageal | 5 (1) |
| Pancreas | 1 (0) |
| Gall Bladder | 1 (0) |
| Colorectal | 18 (3) |
| Parotid | 2 (0) |
| Thyroid | 2 (0) |
| Urothelial | 2 (0) |
| Renal Cell Cancer | 6 (1) |
| Prostate | 2 (0) |
| CML | 1 (0) |
| Melanoma | 28 (4) |
| Ovarian | 1 (0) |
| Germ Cell | 5 (1) |
| Neuroendocrine | 1 (0) |
| Rhabdomyosarcoma | 1 (0) |
| Myxofibrosarcoma | 1 (0) |
| Secondary Lymphoma | 2 (0) |

**Supplementary Table S3 – General Topographic Features:** The relationship of neuroepithelial tumors (NT), primary central nervous system lymphomas (PCNSL) and metastases to the tentorium and the midline are shown. All three entities are more likely to occur above the tentorium. NT however, have a disproportionately increased propensity to supratentorial structures (85%), compared to PCNSL and metastases. Significant right-left-differences were not found in any of the tumor types, but PCNSL were more likely to occur bilaterally. *Abbreviations:* NT: neuroepithelial tumor; PCNSL: primary central nervous system lymphoma; M: metastases.

| **Cerebral Structure** | **Prevalence of Involvement in** | | | ***p* value** | | | |
| --- | --- | --- | --- | --- | --- | --- | --- |
|  | **NT** | **PCNSL** | **M** | **Overall** | **NT vs. PCNSL** | **NT vs. M** | **PCNSL vs. M** |
| **Relationship to Tentorium** |  |  |  |  |  |  |  |
| Supratentorial (only) | 357 (85) | 16 (57) | 140 (70) | < 0.001* | < 0.001* | < 0.001* | 0.117 |
| Infratentorial (only) | 45 (11) | 2 (7) | 23 (11) |  |  |  |  |
| Both | 17 (4) | 10 (36) | 38 (19) |  |  |  |  |
| **Midline Relationship** |  |  |  |  |  |  |  |
| Right (only) | 173 (41) | 4 (14) | 72 (36) | < 0.001* | < 0.001* | 0.142 | 0.003* |
| Left (only) | 149 (36) | 6 (21) | 68 (34) |  |  |  |  |
| Both | 97 (23) | 18 (64) | 61 (30) |  |  |  |  |

**Supplementary Table S4 – Topographic Anatomy of Infratentorial Brain Tumors:** Absolute and relative prevalence of invasion of infratentorial structures in neuroepithelial tumors, primary central nervous system lymphomas and metastases is shown. N = 135. *Abbreviations:* NT: neuroepithelial tumor; PCNSL: primary central nervous system lymphoma; M: metastases; WM: white matter.

| **Cerebral Structure** | **Prevalence of Involvement in** | | | **p value** | | | |
| --- | --- | --- | --- | --- | --- | --- | --- |
|  | **NT**  **N = 62** | **PCNSL**  **N = 12** | **M**  **N = 61** | **Overall** | **NT vs. PCNSL** | **NT vs. M** | **PCNSL vs. M** |
| **BRAINSTEM** |  |  |  |  |  |  |  |
| **Mesencephalon** | 15 (24) | 8 (67) | 0 (0) | < 0.001* | 0.004* | < 0.001* | < 0.001* |
| Central / Aqueduct | 13 (21) | 4 (33) | 0 (0) | < 0.001* | 0.351 | < 0.001* | < 0.001* |
| Tectum | 0 (0) | 2 (17) | 0 (0) | < 0.001* | 0.002* | - | 0.002* |
| Tegmentum | 1 (2) | 0 (0) | 0 (0) | 0.553 | - | - | - |
| Crus | 1 (2) | 3 (25) | 0 (0) | < 0.001* | 0.002* | 0.319 | < 0.001* |
| **Pons** | 4 (6) | 1 (8) | 3 (5) | 0.875 | - | - | - |
| Tegmentum | 4 (6) | 1 (8) | 3 (5) | 0.875 | - | - | - |
| Base | 4 (6) | 1 (8) | 3 (5) | 0.875 | - | - | - |
| **Medulla oblongata** | 2 (3) | 1 (8) | 2 (3) | 0.673 | - | - | - |
| Tegmentum | 2 (3) | 1 (8) | 2 (3) | 0.673 | - | - | - |
| Base | 2 (3) | 1 (8) | 2 (3) | 0.673 | - | - | - |
| **CEREBELLUM** | 34 (55) | 5 (42) | 58 (95) | < 0.001* | 0.403 | < 0.001* | < 0.001* |
| ***Lobes*** |  |  |  |  |  |  |  |
| *Unilobar* | *21 (68)* | *3 (75)* | *50 (89)* | *0.045* | *-* | *-* | *-* |
| *Multilobar* | *10 (32)* | *1 (25)* | *6 (11)* |  |  |  |  |
| Anterior | 9 (15) | 1 (8) | 9 (15) | 0.835 | - | - | - |
| Middle | 8 (13) | 2 (17) | 14 (23) | 0.344 | - | - | - |
| Posterior | 16 (26) | 3 (25) | 40 (66) | < 0.001* | 0.953 | < 0.001* | 0.018 |
| Flocculonodular | 14 (23) | 1 (8) | 1 (2) | 0.001* | 0.388 | 0.001* | 0.388 |
| ***Vermis*** | 18 (29) | 2 (17) | 2 (3) | 0.019 |  |  |  |
| Central | 6 (10) | 1 (8) | 1 (2) | 0.157 | - | - | - |
| Culmen | 5 (8) | 1 (8) | 1 (2) | 0.241 | - | - | - |
| Declive | 4 (6) | 2 (17) | 1 (2) | 0.083 | - | - | - |
| Folium | 4 (6) | 2 (17) | 2 (3) | 0.194 | - | - | - |
| Tuber | 4 (6) | 2 (17) | 1 (2) | 0.083 | - | - | - |
| Pyramid | 4 (6) | 1 (8) | 1 (2) | 0.342 | - | - | - |
| Uvula | 8 (13) | 1 (8) | 1 (2) | 0.058 | - | - | - |
| Nodule | 13 (21) | 1 (8) | 1 (2) | 0.003* | 0.388 | 0.003* | 0.388 |
| ***Hemisphere*** | 17 (27) | 4 (33) | 57 (93) | < 0.001* | 0.677 | < 0.001* | < 0.001* |
| Ala lobuli centralis | 4 (6) | 1 (8) | 3 (5) | 0.875 | - | - | - |
| Anterior Quadrangular | 6 (10) | 1 (8) | 4 (7) | 0.819 | - | - | - |
| Posterior Quadrangular | 4 (6) | 4 (33) | 3 (5) | 0.004* | 0.012 | 0.714 | 0.007* |
| Superior Semilunar | 6 (10) | 3 (25) | 15 (25) | 0.076 | - | - | - |
| Inferior Semilunar / Gracile | 6 (10) | 3 (25) | 33 (54) | < 0.001* | 0.137 | < 0.001* | 0.131 |
| Biventer | 7 (11) | 2 (17) | 11 (18) | 0.565 | - | - | - |
| Tonsilla | 3 (5) | 1 (8) | 3 (5) | 0.876 | - | - | - |
| Flocculus | 3 (5) | 1 (8) | 1 (2) | 0.433 | - | - | - |
| ***Cortical Involvement*** | 31 (50) | 3 (25) | 57 (93) | < 0.001* | 0.122 | < 0.001* | < 0.001* |
| ***WM Sector Involvement*** | 28 (45) | 4 (33) | 56 (92) | < 0.001* | 0.449 | < 0.001* | < 0.001* |
| Subcortical | 28 (45) | 4 (33) | 56 (92) | < 0.001* | 0.449 | < 0.001* | < 0.001* |
| Sublobular | 28 (45) | 4 (33) | 44 (72) | 0.003* | 0.449 | 0.007* | 0.019 |
| Lobular | 27 (44) | 3 (25) | 33 (54) | 0.145 | - | - | - |
| Lobar | 25 (40) | 3 (25) | 4 (7) | < 0.001* | 0.316 | < 0.001* | 0.095 |
| ***Cerebellar Nuclei*** | 4 (6) | 4 (33) | 1 (2) | < 0.001* | 0.012 | 0.177 | < 0.001* |
| ***Cerebellar Peduncles*** | 9 (15) | 4 (33) | 2 (3) | 0.005* | 0.117 | 0.058 | 0.002* |
| Superior | 4 (6) | 2 (17) | 0 (0) | 0.022 | - | - | - |
| Middle | 8 (13) | 3 (25) | 2 (3) | 0.033 | - | - | - |
| Inferior | 5 (8) | 1 (8) | 1 (2) | 0.241 | - | - | - |
| **4^th^ VENTRICLE** | 46 (72) | 4 (33) | 4 (7) | < 0.001* | 0.011 | < 0.001* | 0.011 |
| *Unisegmental* | *34 (74)* | *2 (50)* | *2 (50)* | *0.393* | *-* | *-* | *-* |
| *Multisegmental* | *12 (26)* | *2 (50)* | *2 (50)* |  |  |  |  |
| Superior / Apex | 11 (18) | 2 (17) | 1 (2) | 0.010* | 0.929 | 0.008* | 0.033 |
| Lateral / Recess | 37 (60) | 2 (17) | 2 (3) | < 0.001* | 0.013 | < 0.001* | 0.062 |
| Inferior / Obex | 12 (19) | 2 (17) | 1 (2) | 0.006* | 0.828 | 0.004* | 0.033 |
| Dorsal / Fastigium | 17 (27) | 4 (33) | 2 (3) | < 0.001* | 0.677 | 0.001* | 0.001* |

**Supplementary Table S5 – Prevalence of Metastases in Relation to the Arterial Supply Pattern:** The prevalence of metastases in supra- and infratentorial structures in relation to the pattern of arterial supply (single arterial supply vs. watershed area) are shown. The prevalence of metastases is significantly higher in structures lying at watershed areas, both supra- and infratentorially. *Abbreviations:* ACA: anterior cerebral artery; MCA: middle cerebral artery; PCA: posterior cerebral artery; SCA: superior cerebral artery; AICA: anterior inferior cerebral artery; PICA: posterior inferior cerebral artery.

| **Supratentorial** |  |  |  |
| --- | --- | --- | --- |
| **Single arterial supply** | **Prevalence of Metastases** | **Watershed areas** | **Prevalence of Metastases** |
| Frontal pole (ACA) | 2% | Superior frontal gyrus | 17% |
| Subcallosal area (ACA) | 0% | Middle frontal gyrus | 19% |
| Gyrus rectus (ACA) | 0% | Precentral gyrus | 14% |
| Rostral gyrus (ACA) | 1% | Postcentral gyrus | 9% |
| Paracentral lobule (ACA) | 3% | Superior parietal lobule | 8% |
| Inferior frontal gyrus (MCA) | 5% | Inferior parietal lobule | 13% |
| Subcentral gyrus (MCA) | 3% | Precuneus | 7% |
| Superior temporal gyrus (MCA) | 3% | Middle occipital gyrus | 8% |
| Temporal pole (MCA) | 1% |  |  |
| Short insular gyri (MCA) | 0% |  |  |
| Long insular gyri (MCA) | 1% |  |  |
| Parahippocampal gyrus (PCA) | 0% |  |  |
| Occipital pole (PCA) | 1% |  |  |
| Superior occipital gyrus (PCA) | 3% |  |  |
| Inferior occipital gyrus (PCA) | 3% |  |  |
| Cuneus (PCA) | 4% |  |  |
| **Infratentorial** |  |  |  |
| **Single arterial supply** | **Prevalence of Metastases** | **Watershed areas** | **Prevalence of Metastases** |
| Ala lobuli centralis (SCA) | 5% | Superior semilunar lobule | 25% |
| Anterior quadrangular lobule (SCA) | 7% | Inferior semilunar/gracile lobule | 54% |
| Posterior quadrangular lobule (SCA) | 5% | Biventer lobule | 18% |
| Superior vermis (SCA) | 2-3% |  |  |
| Flocculus (AICA) | 2% |  |  |
| Inferior vermis (PICA) | 2-3% |  |  |
| Tonsil (PICA) | 5% |  |  |
